# Machine Learning-Based Non-Invasive Prediction of Metabolic Dysfunction-Associated Steatohepatitis in Obese Patients: A Retrospective Study

**DOI:** 10.3390/diagnostics15091096

**Published:** 2025-04-25

**Authors:** Jie Chen, Bo Zhang, Yong Cheng, Yuanchen Jia, Biao Zhou

**Affiliations:** 1 Department of Ultrasound, China-Japan Friendship Hospital, Beijing 100029, China; 2 School of Information Science and Technology, Beijing University of Chemical Technology, Beijing 100029, China; 3 Department of General Surgery & Obesity and Metabolic Disease Center, China-Japan Friendship Hospital, Beijing 100029, China

**Keywords:** metabolic dysfunction-associated steatohepatitis (MASH), metabolic dysfunction-associated fatty liver disease (MAFLD), machine learning, non-invasive diagnosis

## Abstract

**Objectives**: We aimed to develop and validate machine learning (ML) models that integrate clinical and laboratory data for the non-invasive prediction of metabolic dysfunction-associated steatohepatitis (MASH) in an obese population. **Methods**: In this retrospective study, clinical and laboratory data were collected from obese patients undergoing bariatric surgery. The cohort was divided using stratified random sampling, and optimal features were selected with SHapley Additive exPlanations (SHAP). Various ML models, including K-nearest neighbors, linear support vector machine, radial basis function support vector machine, Gaussian process, random forest, multilayer perceptron, adaptive boosting, and naïve Bayes, were developed through cross-validation and hyperparameter tuning. Diagnostic performance was assessed via the area under the curve (AUC) in both training and validation sets. **Results**: A total of 558 patients were analyzed, with 390 in the training set and 168 in the validation set. In the training cohort, the median age was 35 years, the median body mass index (BMI) was 39.8 kg/m^2^, 39.0% were male, 37.9% had diabetes mellitus, and 62.8% were diagnosed with MASH. The validation cohort had a median age of 34.1 years, a median BMI of 42.5 kg/m^2^, 41.7% male, 32.7% with diabetes, and 39.9% with MASH. Among the models, the random forest achieved the highest performance among the models with AUC values of 0.94 in the training set and 0.88 in the validation set. The Gaussian process model attained an AUC of 0.97 in the training cohort but 0.79 in the validation cohort, while the other models achieved AUC values ranging from 0.63 to 0.88 in the training cohort and 0.62 to 0.75 in the validation set. **Conclusions**: ML models, particularly the random forest, effectively predict MASH using readily available data, offering a promising non-invasive alternative to conventional serological scoring. Prospective studies and external validations are needed to further establish clinical utility.

## 1. Introduction

Non-alcoholic fatty liver disease (NAFLD), one of the most prevalent chronic liver diseases worldwide, affects approximately 25% of the global population, with this figure increasing annually. Changes in lifestyle and the increasing prevalence of metabolic syndrome in recent decades have significantly escalated the global burden of NAFLD [1]. In China, the prevalence of NAFLD has surged over the past decade to nearly 30%, a trend closely associated with rapid urbanization and consequent metabolic disturbances [2]. The progression of NAFLD is multifaceted; simple non-alcoholic fatty liver can advance to non-alcoholic steatohepatitis (NASH), characterized by inflammation and hepatocellular injury, which may further progress to fibrosis and eventually cirrhosis [3]. Both NASH and fibrosis are strongly associated with impaired liver function and a heightened risk of liver-related complications, including cirrhosis, liver failure, and hepatocellular carcinoma [4]. In recognition of the limitations of the NAFLD nomenclature in reflecting the underlying pathogenesis and heterogeneity, the term metabolic dysfunction-associated fatty liver disease (MAFLD) has been introduced. This updated classification better captures the metabolic foundations of the disease and aims to improve patient stratification and facilitate drug development [5]. Accordingly, NASH has been renamed metabolic dysfunction-associated steatohepatitis (MASH), highlighting the necessity for early identification of high-risk patients to enable timely intervention and improved clinical outcomes [6].

Liver biopsy remains the gold standard for diagnosing MAFLD and its various pathological stages. However, due to its invasive nature, susceptibility to sampling errors, patient discomfort, and high costs, liver biopsy has significant limitations in routine clinical practice [7,8]. Traditional non-invasive scoring models, such as the Fibrosis-4 (FIB-4) index and the MAFLD fibrosis score, are commonly employed to predict liver fibrosis. Yet their accuracy varies among different patient populations, and they demonstrate limited diagnostic capability for MASH [9,10]. For instance, although the FIB-4 index offers valuable non-invasive insights into liver fibrosis, it demonstrates poor sensitivity in detecting mild fibrosis and may misclassify patients who also have inflammatory or extrahepatic conditions [11].

Recent advances in machine learning (ML) have made it increasingly feasible to develop predictive models that overcome the limitations of traditional non-invasive assessments, offering promising new tools for forecasting MAFLD progression [12,13]. By mining complex, large-scale datasets for latent patterns, ML can markedly enhance the prediction of disease status and progression. For example, studies have shown that ML models based on random forest (RF) algorithms achieved area under the curve (AUC) values of 0.89 for both significant fibrosis and cirrhosis (≥F2 and F4), outperforming both FibroScan and FIB-4 [14]. Moreover, Newsome et al. demonstrated that the FibroScan-AST (aspartate aminotransferase) score effectively identifies patients with MASH and advanced fibrosis [15]. Furthermore, a deep learning system developed in a Chinese community cohort achieved AUC values of 0.90 and 0.93 in detecting the presence and severity of MAFLD, respectively, surpassing traditional blood biomarker indices [16].

Although numerous advanced ML techniques, such as hybrid or optimized models, are increasingly explored in various applied domains [17,18,19], established ML algorithms, such as RF and naïve Bayes, and others still hold significant advantages in terms of interpretability, clinical applicability, and proven robustness in similar clinical contexts [20,21]. These advantages assist established ML algorithms in translating their predictions into practical therapeutic interventions more easily. To date, few studies have directly utilized interpretable ML models specifically designed to predict biopsy-proven MASH by integrating comprehensive clinical and laboratory parameters in obese populations.

In the study we presented herein, we aimed to develop an ML-based predictive model for MASH using histopathology as the gold standard. Our approach integrates comprehensive clinical with laboratory test results to assess the model’s predictive performance and explore its potential clinical applications. Ultimately, our goal is to provide clinicians with a robust, interpretable, and clinically practical diagnostic tool, facilitating the early identification and personalized clinical management of MASH.

## 2. Materials and Methods

### 2.1. Study Design and Participants

We have collected liver pathology data, clinical records, and blood laboratory test results from inpatients who underwent bariatric surgery at the Obesity and Metabolic Disease Center of China–Japan Friendship Hospital between 2020 and July 2024. All clinical and laboratory data were obtained within one month before collecting liver tissue samples. Inclusion criteria were (a) patients undergoing bariatric surgery and (b) availability of complete data on liver pathology, clinical information, and blood laboratory tests. Exclusion criteria were (a) the presence of hepatocellular carcinoma (HCC) or other malignancies; (b) any end-stage disease or systemic inflammatory conditions resulting from persistent infections, respiratory failure, decompensated heart failure, or flares of autoimmune diseases; (c) hepatitis B or hepatitis C infection; (d) alcohol-related fatty liver disease, defined as ethanol consumption of ≥30 g per day for men and ≥20 g per day for women within the past 12 months; (e) drug-induced liver disease or autoimmune hepatitis; and (f) incomplete relevant data. This study was registered with the Chinese Clinical Trial Registry (ChiCTR2300078083) and received approval from the Institutional Ethics Committees of the participating hospital (approval number 2023-KY-203-2).

### 2.2. Liver Pathology

Liver tissue samples were obtained intraoperatively during bariatric surgery. The decision to collect liver tissue was made by the operating surgeon based on the patient’s metabolic syndrome status, and written informed consent was obtained from each patient prior to the procedure. A small wedge of liver tissue, approximately 1 cm at its longest dimension, was excised from the left lobe. Experienced pathologists performed all histopathological evaluations. Steatosis was graded as follows: score 0 (<5% hepatocyte involvement), score 1 (5–33%), score 2 (33–66%), and score 3 (>66%). Fibrosis was staged as follows: Stage 0 (no fibrosis), Stage 1 (zone 3 perisinusoidal fibrosis), Stage 2 (zone 3 perisinusoidal fibrosis combined with portal fibrosis), Stage 3 (bridging fibrosis), and Stage 4 (cirrhosis). The NAFLD Activity Score was utilized to assess MASH by combining scores for steatosis (0–3), lobular inflammation (0–3), and hepatocellular ballooning (0–2), yielding a total score ranging from 0 to 8 [22].

### 2.3. Statistical Analysis

Patient data were classified as either continuous or categorical variables. Data with a normal distribution are presented as mean ± standard deviation, whereas non-normally distributed data are expressed as medians with interquartile ranges. Group comparisons for continuous variables were conducted using independent-samples *t*-tests for normally distributed data and Mann–Whitney U tests for nonparametric data. Categorical variables were compared using Pearson’s chi-square test or Fisher’s exact test when expected cell counts were less than five. Diagnostic accuracy of the models was evaluated by AUC. Optimal cut-off values were determined by maximizing sensitivity and specificity using Youden’s index. Univariable and multivariable logistic regression analyses were performed to assess risk factors associated with MASH. All statistical tests were two-sided, with a significance level set at *p* < 0.05. Analyses were performed using SPSS software (version 25.0, IBM Corporation, Armonk, NY, USA).

### 2.4. Machine Learning Data Processing

A comprehensive data preprocessing protocol was implemented. Samples with more than 20% missing values were systematically removed. Statistical outliers were identified using Mahalanobis distance (*p* < 0.001). All numerical features were standardized to a mean of zero and a unit variance to ensure algorithmic stability and facilitate fair feature comparison. Eight machine learning algorithms were evaluated: K-nearest neighbors (KNNs), linear support vector machine (linear SVM), radial basis function support vector machine (RBF SVM), adaptive boosting (AdaBoost), Gaussian process (GP), RF, multilayer perceptron (MLP), and naïve Bayes. The eight models encompass a variety of machine learning methods, ranging from simple to complex and from linear to nonlinear, including both supervised and probabilistic models. In this study, different machine learning algorithms utilized features in distinct ways. The KNN algorithm used all available features to compute distances between samples without performing any feature selection, making it particularly sensitive to noise. The linear SVM assigned weight coefficients to each feature, with larger weights indicating higher importance. In contrast, the RBF SVM model mapped features into a high-dimensional space using a non-linear kernel, making individual feature importance difficult to interpret. The GP model treated all features equally and did not perform explicit feature selection. RF model inherently performs feature selection by splitting nodes based on information gain or Gini impurity, thus highlighting informative features. The MLP model accepted all input features, learning weights through backpropagation; although certain features might influence predictions more strongly, the model did not provide direct feature importance scores. The AdaBoost model selected the most informative features iteratively by focusing on misclassified samples in each boosting round. Lastly, the naïve Bayes classifier assumed conditional independence among features and incorporated all of them equally into the probabilistic model.

The dataset was stratified and partitioned into training and validation sets using stratified random sampling to maintain the original class distribution across groups. To ensure methodological rigor and reproducibility, all data partitioning procedures were conducted with a predefined random seed, ensuring consistency across experiments. Models were optimized employing a nested cross-validation strategy, comprising an outer five-fold cross-validation for performance assessment and an inner five-fold cross-validation for hyperparameter tuning via grid search. Model performance was evaluated using multiple metrics, including the AUC, accuracy, sensitivity, specificity, and precision-recall curves.

## 3. Results

### 3.1. Patient Characteristics

A total of 569 patients who underwent bariatric surgery with concurrent liver biopsies were initially identified. After excluding 11 patients with incomplete data, 558 patients were included in the final analysis. The cohort was then divided into a training set (*n* = 390) and a validation set (*n* = 168) by stratified random sampling. Baseline characteristics for both cohorts are summarized in Table 1. In the training cohort, the median age was 35.0 years, with a median body mass index (BMI) of 39.8 kg/m^2^; 152 patients (39.0%) were male, and 37.9% had diabetes mellitus (DM). In the validation cohort, the median age was 34.1 years, the median BMI was 42.5 kg/m^2^, with 41.7% male and 32.7% diagnosed with DM. The relevant baseline characteristics of the study population are shown in Table 1.

### 3.2. Univariate and Multivariate Analyses

A detailed summary of the univariate analysis results identifying factors associated with the occurrence of MASH is provided in Table 2. Factors including hypertension, diabetes mellitus, body weight, BMI, ALT, AST, albumin, triglycerides, fasting insulin, HDL-C, steatosis grade, and fibrosis stage were significantly associated with MASH (all *p* < 0.05). In contrast, sex, age, height, platelet count, total bilirubin, uric acid, total cholesterol, LDL-C, creatinine, and glycated hemoglobin showed no significant association (all *p* > 0.05).

When we applied LASSO (Least Absolute Shrinkage and Selection Operator) regression to select features/variables before performing the multivariate analysis, only a subset of variables in such obtained multivariate model remained statistically significant. Specifically, BMI (*p* = 0.0498, OR = 1.248, 95% CI: 1.000–1.557), ALT (*p* = 0.002, OR = 1.409, 95% CI: 1.128–1.759), and TG (*p* = 0.029, OR = 1.261, 95% CI: 1.025–1.553) remained significantly associated with MASH. In contrast, some factors significant in the univariate analysis, such as HDL-C and ALB, did not retain significance in the multivariate model, probably because other variables confounded these associations. Similarly, variables that were non-significant in the univariate analysis (e.g., total bilirubin, uric acid) remained non-significant in the multivariate analysis, indicating a weak or absent relationship with MASH.

### 3.3. Diagnostic Performance of ML Models for MASH

To develop predictive models for diagnosing MASH, 20 variables from the training cohort were integrated into several machine learning algorithms. SHAP values were then employed to quantify the contribution of each variable to the model. In the KNN model, the final features included PLT, uric acid, ALT, body weight, creatinine, fasting insulin, AST, age, height, BMI, total bilirubin, HDL-C, triglycerides (TG), LDL-C, sex, DM, hypertension, ALB, TC, and HbA1c.

For the linear SVM model, the selected variables were BMI, body weight, height, ALT, age, AST, creatinine, uric acid, fasting insulin, DM, HbA1c, PLT, hypertension, HDL-C, TC, TG, ALB, LDL-C, sex, and total bilirubin. Similarly, the RBF SVM model utilized ALT, uric acid, body weight, BMI, height, PLT, fasting insulin, total bilirubin, ALB, HbA1c, age, AST, creatinine, TG, LDL-C, DM, sex, TC, HDL-C, and hypertension. The GP model was constructed using the following variables: ALT, body weight, PLT, height, age, uric acid, creatinine, BMI, total bilirubin, AST, TG, ALB, fasting insulin, DM, HbA1c, LDL-C, TC, HDL-C, sex, and hypertension. The RF model comprised ALT, DM, age, HDL-C, ALB, TG, fasting insulin, AST, LDL-C, creatinine, body weight, PLT, height, HbA1c, total bilirubin, hypertension, BMI, TC, uric acid, and sex.

**Figure 1:**
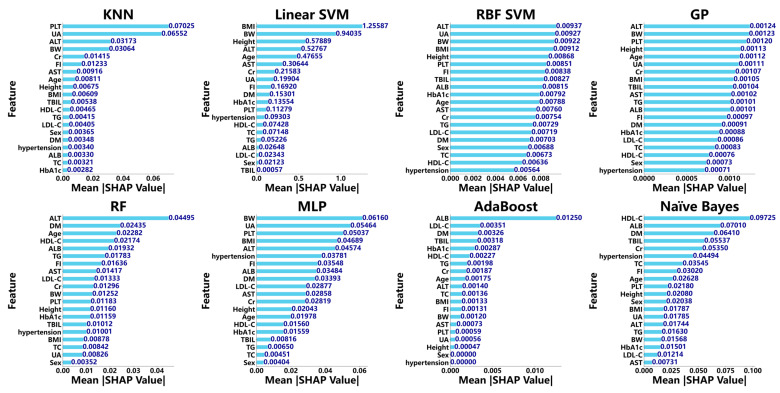
SHapley Additive exPlanations (SHAP) plot illustrating the contribution of clinical features in diagnosing MASH across multiple machine learning models, including K-nearest neighbors (KNNs), linear support vector machine (linear SVM), radial basis function support vector machine (RBF SVM), adaptive boosting (AdaBoost), Gaussian process (GP), random forest (RF), multilayer perceptron (MLP), adaptive boosting (AdaBoost), and naïve Bayes. Note: BMI, body mass index; PLT, platelet count; TBIL, total bilirubin; ALB, albumin; ALT, alanine transaminase; AST, aspartate transaminase; UA, uric acid; TC, total cholesterol; TG, triglycerides; BW, body weight; FI, fasting insulin; Cr, creatinine; HDL-C, high-density lipoprotein cholesterol; LDL-C, low-density lipoprotein cholesterol; DM, diabetes mellitus; HbA1c, glycated hemoglobin.

In the MLP model, the final features were body weight, uric acid, PLT, BMI, ALT, hypertension, fasting insulin, ALB, DM, LDL-C, AST, creatinine, height, age, HDL-C, HbA1c, total bilirubin, TG, TC, and sex. The AdaBoost model was built using ALB, LDL-C, DM, total bilirubin, HbA1c, HDL-C, TG, creatinine, age, ALT, TC, BMI, fasting insulin, body weight, AST, PLT, uric acid, height, sex, and hypertension. The naïve Bayes model incorporated HDL-C, ALB, DM, total bilirubin, creatinine, hypertension, TC, fasting insulin, age, PLT, height, sex, BMI, uric acid, ALT, TG, body weight, HbA1c, LDL-C, and AST. The compositions of the aforementioned models are illustrated in Figure 1.

**Figure 2:**
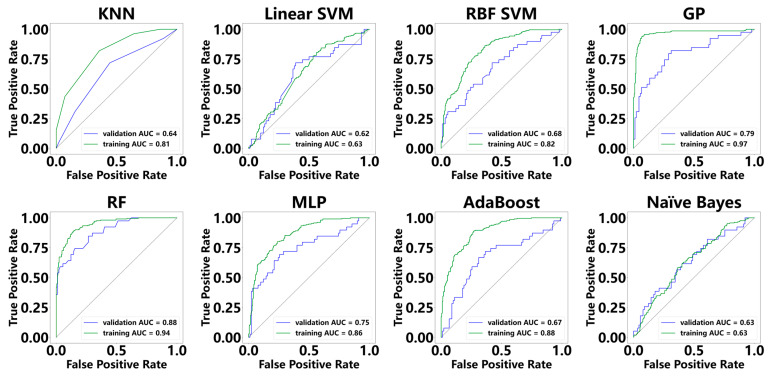
Diagnostic performance of machine learning models for diagnosing MASH in the training cohort and the validation cohort. Note: K-nearest neighbors (KNNs); linear support vector machine (linear SVM); radial basis function support vector machine (RBF SVM); Gaussian process (GP); random forest (RF); multilayer perceptron (MLP); adaptive boosting (AdaBoost).

In the training cohort, the AUC values of these models ranged from 0.63 to 0.97, whereas in the validation cohort, they varied from 0.62 to 0.88 (Figure 2). The GP model attained the highest AUC in the training cohort (AUC = 0.97); its performance declined in the validation cohort, where it recorded an AUC of 0.79 and ranked second among the models. Notably, the RF model demonstrated the highest performance in the validation cohort, achieving an AUC of 0.88, and in the training cohort, it achieved an AUC of 0.94. For the remaining models, KNN, linear SVM, RBF SVM, MLP, AdaBoost, and naïve Bayes, the AUC values in the training cohort were 0.81, 0.63, 0.82, 0.86, 0.88, and 0.63, respectively, and in the validation cohort, they were 0.64, 0.62, 0.68, 0.75, 0.67, and 0.63.

**Figure 3:**
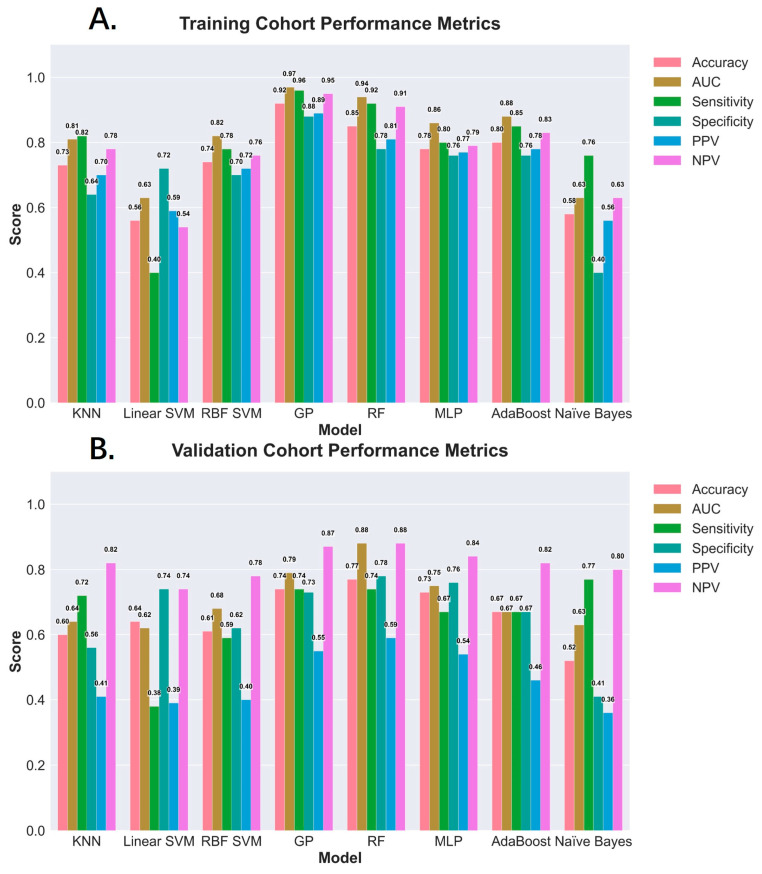
Diagnostic performance of machine learning models for diagnosing MASH in the training cohort (**A**) and the validation cohort (**B**). Note: K-nearest neighbors (KNNs); linear support vector machine (linear SVM); radial basis function support vector machine (RBF SVM); Gaussian process (GP); random forest (RF); multilayer perceptron (MLP); adaptive boosting (AdaBoost).

In the training cohort, the GP model achieved an accuracy of 0.92, an AUC of 0.97, a sensitivity of 0.96, a specificity of 0.88, a positive predictive value (PPV) of 0.89, and a negative predictive value (NPV) of 0.95. The corresponding values in the validation cohort were 0.74, 0.79, 0.74, 0.73, 0.55, and 0.87. Meanwhile, the RF model demonstrated an accuracy of 0.85, an AUC of 0.94, a sensitivity of 0.92, a specificity of 0.78, a PPV of 0.81, and an NPV of 0.91 in the training cohort, and achieved 0.77, 0.88, 0.74, 0.78, 0.59, and 0.88, respectively, in the validation cohort (Figure 3A,B) (see Supplementary Table S1 for the tabular format).

**Figure 4:**
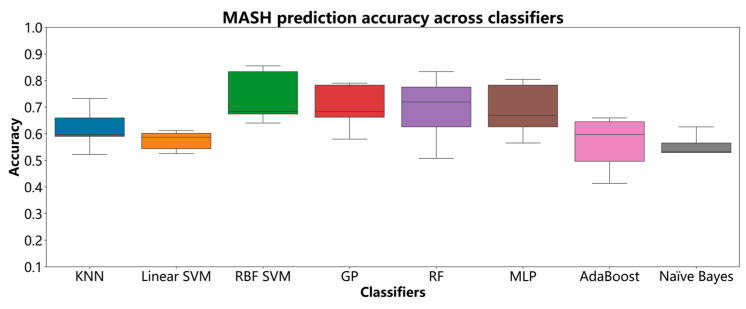
MASH prediction accuracy across classifiers.

Figure 4 presents boxplots of model accuracy, calculated by comparing predicted values with true outcomes, derived from the cross-validation of eight classifiers. As shown, the prediction accuracies for the KNN, linear SVM, RBF SVM, GP, RF, MLP, AdaBoost, and naïve Bayes models were compared across multiple experiments. The results indicate that the RBF SVM and GP models achieved the best performance for this task, with median accuracies of 0.74 and 0.70, respectively. Additionally, the MLP and RF models both achieved median accuracies of 0.69. In contrast, the KNN, linear SVM, AdaBoost, and naïve Bayes models exhibited lower accuracies, with median values of 0.62, 0.57, 0.56, and 0.56, respectively.

### 3.4. Transaminase Performance in Diagnosing MASH

The AUC for diagnosing MASH using elevated ALT/AST levels (ALT levels above 40 U/L or AST levels above 42 U/L) was 0.572. The PPV was 0.455 (122/(122 + 146)), and the NPV was 0.690 (200/(200 + 90)). Additionally, the false positive rate was 0.545 (146/(146 + 122)), and the false negative rate was 0.310 (90/(90 + 200)).

## 4. Discussion

In this study, we focused on an obese population, where MAFLD often follows an accelerated course toward MASH. We employed ML methods to establish a predictive model that integrates clinical information and blood laboratory tests for the assessment of MASH. The eight models employed in this study represent a broad spectrum of ML approaches, ranging from simple to complex architectures, linear to nonlinear formulations, and encompassing both supervised and probabilistic frameworks. The KNN algorithm classifies a sample by computing its distances to all samples in the training set, selecting the K nearest ones, and determining the final class through majority voting based on their labels [23,24]. Support vector machine (SVM) is a margin-based classification technique widely used in ML. The linear SVM, which applies a linear kernel, is particularly effective for high-dimensional data scenarios [25]. The RBF SVM algorithm extends this concept by projecting data into a higher-dimensional feature space, improving class separability for non-linearly distributed data [26]. The GP algorithm is a non-parametric Bayesian approach based on multivariate Gaussian distributions. It performs probabilistic function modeling and provides uncertainty estimation for each prediction, which is particularly valuable in clinical decision-making contexts [27,28]. The RF algorithm is a combination of tree predictors such that each tree depends on the values of a random vector sampled independently and with the same distribution for all trees in the forest. As the number of trees increases, the generalization error tends to converge to a stable limit [29,30]. The MLP model is a feedforward artificial neural network that maps sets of input data onto a set of appropriate outputs. It is a modification of the standard linear perceptron in that it uses three or more layers of neurons (nodes) with nonlinear activation functions [31]. AdaBoost algorithms combine multiple weak classifiers to generate a strong classifier by adaptively determining the fusion weights of the weak classifiers [32]. The naïve Bayes classifier greatly simplifies learning by assuming that features are independent given class [33].

The ML models we developed, specifically the RF and GP models, demonstrated excellent predictive performance in the validation cohort. The RF model achieved AUC values of 0.94 and 0.88 in the training and validation cohorts, respectively, while the GP model yielded AUC values of 0.97 in the training cohort and 0.79 in the validation cohort. In our study population, elevated ALT/AST levels yielded an AUC of 0.572, with an NPV of 0.690 and a false negative rate of 0.310. The strong performance of the GP and RF models likely stems from their inherent strengths. The GP model offers uncertainty quantification for each prediction, allowing a better balance between bias and variance. It is particularly robust to noise and well-suited for small datasets with complex features, as often seen in clinical settings. The RF model effectively captures nonlinear relationships and includes an embedded feature selection mechanism, which enhances its resistance to overfitting. In this study, limiting tree depth (max_depth = 5) reduced model complexity and improved generalizability. These characteristics contribute to their reliable performance in medical datasets of moderate size. Although the RBF SVM model achieved the highest accuracy, its AUC was not the best among all models, suggesting potential limitations in its ability to identify positive cases.

In clinical practice, liver biopsy is not routinely used for screening; it is generally performed when MAFLD patients exhibit elevated transaminases indicative of liver injury, which may then prompt the initiation of hepatoprotective treatment. ALT reduction correlates with histological improvement, and ALT normalization can predict NASH resolution in response to lifestyle modification as well as various therapeutic interventions [34]. The RF and GP models show potential in helping clinicians identify MASH, evaluate disease severity, plan further diagnostic workup, and inform treatment decisions. In contrast, machine learning models offer the potential to diagnose MASH early before significant liver injury occurs.

Obesity is a well-established risk factor for the onset and progression of MASH. Even individuals classified as metabolically healthy but obese exhibit an increased risk of developing MAFLD and MASH, indicating that obesity itself can lead to subclinical liver disease [35]. The pathogenesis of MASH in obese patients is driven, in part, by inflammatory responses and oxidative stress, which also elevate the risk of HCC. The “multiple-hit hypothesis” has been proposed to account for these complex pathological processes [36]. Bariatric surgery has emerged not only as an effective intervention for reducing obesity but also as a way to significantly decrease the incidence of both MASH and HCC, thereby conferring long-term hepatic benefits in severely obese individuals [37]. Notably, a previous study reported that 33.6% of morbidly obese patients undergoing bariatric surgery were diagnosed with MASH, with 31.3% demonstrating fibrosis, underscoring the critical influence of metabolic factors in disease progression [38]. These findings emphasize that early identification and intervention are paramount to prevent progression to cirrhosis and HCC. In 2024, the U.S. FDA approved Resmetirom, the world’s first drug specifically indicated for the treatment of MASH. Resmetirom is a liver-targeted, thyroid hormone receptor-β selective agonist that has shown promise in promoting the regression of MASH and ameliorating liver fibrosis [39]. Thus, the early detection of MASH is essential to enable timely therapeutic intervention and improve patient outcomes.

Non-invasive methods play a critical role in diagnosing and managing MAFLD, with serological assessments being particularly valuable. For example, the MASH test combines demographic characteristics (age, sex, and BMI), serum parameters (ALT, AST, and lipids), and specific biomarkers such as alpha-2 macroglobulin, apolipoprotein A1, and haptoglobin. This test exhibits a sensitivity of 33% and a specificity of 94%, resulting in a robust NPV of 81% for MASH [40]. In a study involving 257 patients who underwent liver biopsy, the AUCs for diagnosing MASH, borderline MASH, and no MASH were 0.79, 0.69, and 0.77, respectively [41]. Additionally, Fibrosis-4 (Fib-4) remains a frontline tool for screening liver fibrosis and assessing its severity [42]. However, while the Fib-4 index serves as a non-invasive method for evaluating liver fibrosis and predicting related events such as HCC, its diagnostic accuracy is somewhat limited, with an accuracy of only 0.76 reported for detecting advanced fibrosis in patients with NAFLD [43].

With the rapid development of ML, clinicians can now leverage large datasets to build predictive models that enhance diagnostic accuracy. Numerous studies have applied ML to the assessment of NAFLD. For instance, Lee et al. reported that clinical Gradient Boosting machine models achieved AUCs of 0.94, 0.79, and 0.72 for predicting steatosis, inflammation, and ballooning, respectively; notably, the addition of biomarkers did not further improve model performance [44]. Other ML approaches, such as the MASHmap model, have been used to identify patients with potential MASH from electronic health records, achieving AUCs of 0.82 in the training set and 0.76 in the validation set, thereby assisting clinicians in identifying high-risk patients for targeted diagnostic testing and therapeutic intervention [45]. Furthermore, ML models trained on medical claims data have also demonstrated the capability to detect patients likely to have MASH, facilitating early diagnosis and effective disease management [46]. In our study, the machine learning models we developed, specifically the RF and GP models, demonstrated robust predictive performance in the validation cohort. SHAP analysis identified key predictive features, including ALT, age, HDL-C, DM, and ALB. These variables are routinely available in clinical practice and are cost-effective, enhancing the practical utility of our models.

Nevertheless, our study has several limitations. First, the retrospective design may introduce selection bias, and future studies should employ prospective designs to validate the generalizability of our models across diverse populations. Second, our study cohort was limited to a single region and included only obese patients undergoing bariatric surgery, which may introduce selection bias and restrict the generalizability of the findings to non-surgical obese populations and other regions. Future multicenter studies with broader populations are recommended to improve external validity. Third, MAFLD is a multifactorial disease influenced by genetic predisposition and the presence of comorbidities [5,47]. Integrating these additional variables into ML models could potentially enhance predictive accuracy; however, such detailed information was not available in our retrospective dataset.

## 5. Conclusions

In summary, our study underscores the superior performance of ML-based models for predicting MASH in an obese MAFLD cohort. Looking ahead, ML is poised to become a cornerstone in the precision diagnosis of MASH and may guide further liver biopsy to confirm MASH. ML can facilitate early screening and dynamic monitoring, thereby guiding personalized lifestyle interventions or targeted pharmacotherapy to halt disease progression. As data diversity and model sophistication continue to advance, the approach proposed in this study holds promise for delivering a comprehensive solution for the non-invasive diagnosis and management of MASH.

## Figures and Tables

**Table 1:** Baseline characteristics of the studied cohort.

Characteristics	Training Cohort (*n* = 390)	Validation Cohort (*n* = 168)
Male	152 (39.0%)	70 (41.7%)
Age (years)	34.0 (28.0–41.0)	34.0 (27.8–40.3)
Body weight (kg)	110.0 (95.0–127.8)	114.0 (96.7–130.4)
Height (cm)	168.0 (162.5–174.0)	168.5 (164.0–175.0)
Body mass index (kg/m^2^)	39.0 (34.6–43.4)	39.8 (34.8–44.8)
Diabetes mellitus	148 (37.9%)	55 (32.7%)
Hypertension	153 (39.2%)	64 (38.1%)
Platelet count (×10^9^/L)	260.0 (220.0–303.0)	277 (235.5–310.5)
Alanine transaminase (U/L)	37.0 (22.0–57.8)	39.0 (22.8–63.0)
Aspartate transaminase (U/L)	24.0 (16.0–33.0)	25.0 (18.0–34.0)
Total bilirubin (µmol/L)	12.3 (9.7–15.3)	12.9 (9.5–16.0)
Albumin (g/L)	43.6 (41.7–45)	43.6 (42.0–45.0)
Uric acid (µmol/L)	421.0 (352.0–491.5)	435.0 (356.0–503.3)
Total cholesterol (mmol/L)	4.9 (4.3–5.4)	4.9 (4.4–5.4)
Triglyceride (mmol/L)	1.8 (1.3–2.5)	1.9 (1.3–2.4)
High-density lipoprotein cholesterol (mmol/L)	1.0 (0.8–1.1)	1.0 (0.8–1.1)
Low-density lipoprotein cholesterol (mmol/L)	3.1 (2.6–3.5)	3.1 (2.7–3.6)
Creatinine (μmol/L)	55.7 (47.6–65.6)	58.7 (48.4–66.0)
Glycosylated hemoglobin (%)	6.1 (5.6–6.8)	6.2 (5.6–6.7)
Fasting insulin (pmol/mL)	23.5 (15.4–30.1)	24.1 (17.2–29.1)
Grade of steatosis		
Grade 0	36 (9.2%)	21 (12.5)
Grade 1	157 (40.3%)	66 (39.3%)
Grade 2	125 (32.1%)	44 (26.2%)
Grade 3	72 (18.4%)	37 (22.0%)
MASH: Yes	245 (62.8%)	67 (39.9%)
MASH: No	145 (37.2%)	101 (60.1%)
Fibrosis stage		
F0	309 (79.2%)	126 (75.0%)
F1	58 (14.9%)	35 (20.8%)
F2	8 (2.0%)	3 (1.8%)
F3	12 (3.1%)	3 (1.8%)
F4	3 (0.8)	1 (0.6%)

Note: Values presented as n (%), median (interquartile range).

**Table 2:** Univariate analysis of factors associated with MASH.

Variables	OR (95% CI)	*p*-Value
Hypertension	1.55 (1.06–2.27)	0.025
Diabetes mellitus	1.78 (1.21–2.62)	0.004
Body weight	1.23 (1.02–1.48)	0.028
Body mass index	1.24 (1.03–1.50)	0.021
Alanine aminotransferase	1.51 (1.25–1.84)	<0.001
Aspartate aminotransferase	1.43 (1.18–1.73)	<0.001
Albumin	1.27 (1.05–1.54)	0.012
Triglycerides	1.27 (1.06–1.54)	0.011
Fasting insulin	1.37 (1.13–1.65)	0.001
High-density lipoprotein cholesterol	0.82 (0.67–0.99)	0.038
Steatosis grade	2.03 (1.62–2.53)	<0.001
Fibrosis stage	3.72 (2.47–5.60)	<0.001
Sex	0.78 (0.53–1.14)	0.205
Age	0.98 (0.81–1.18)	0.819
Height	1.10 (0.91–1.32)	0.314
Platelet count	1.09 (0.91–1.31)	0.365
Total bilirubin	0.99 (0.82–1.19)	0.928
Uric acid	1.12 (0.93–1.34)	0.244
Total cholesterol	1.11 (0.92–1.34)	0.269
Low-density lipoprotein cholesterol	1.13 (0.93–1.36)	0.212
Creatinine	1.01 (0.84–1.21)	0.954
Glycated hemoglobin	1.14 (0.95–1.37)	0.156

## Data Availability

The original contributions presented in the study are included in the article; further inquiries can be directed to the corresponding authors.

## Supplementary Materials

The following supporting information can be downloaded at: https://www.mdpi.com/article/10.3390/diagnostics15091096/s1, Table S1: Diagnostic performance of the machine learning models for MASH.

## Abbreviations

The following abbreviations are used in this manuscript:

ML	Machine learning
MASH	Metabolic dysfunction-associated steatohepatitis
SHAP	SHapley Additive exPlanations
AUC	Area under the curve
MAFLD	Metabolic dysfunction-associated fatty liver disease
NAFLD	Non-alcoholic fatty liver disease
NASH	Non-alcoholic steatohepatitis
FIB-4	Fibrosis-4
RF	Random forest
HCC	Hepatocellular carcinoma
KNNs	K-nearest neighbors
Linear SVM	Linear support vector machine
MLP	Multilayer perceptron
AdaBoost	Adaptive boosting
DM	Diabetes mellitus
BMI	Body Mass Index
ALT	Alanine aminotransferase
ALB	Albumin
HDL-C	High-density lipoprotein cholesterol
LDL-C	Low-density lipoprotein cholesterol
PLT	Platelet count
TG	Triglycerides
TC	Triglyceride
PPV	Positive predictive value
NPV	Negative predictive value
