# Supplementary material for: Machine Learning-Based Non-Invasive Prediction of Metabolic Dysfunction-Associated Steatohepatitis in Obese Patients: A Retrospective Study
Source: Diagnostics (Basel). 2025 Apr 25;15(9):1096. doi: 10.3390/diagnostics15091096 (PMC12072127; doi:10.3390/diagnostics15091096)
Supplement: Supplementary file 1 [file diagnostics-15-01096-s001.zip › diagnostics-3552335-supplementary.pdf]

Supplementary Table S1. Diagnostic performance of the machine learning models for MASH.

	Accuracy	AUC	Sensitivity	Specificity	Positive Predictive Value	Negative Predictive Value
Training cohort						
KNN	0.73	0.81	0.82	0.64	0.70	0.78
Linear SVM	0.56	0.63	0.40	0.72	0.59	0.54
RBF SVM	0.74	0.82	0.78	0.70	0.72	0.76
GP	0.92	0.97	0.96	0.88	0.89	0.95
RF	0.85	0.94	0.92	0.78	0.81	0.91
MLP	0.78	0.86	0.80	0.76	0.77	0.79
AdaBoost	0.80	0.88	0.85	0.76	0.78	0.83
Naïve Bayes	0.58	0.63	0.76	0.40	0.56	0.63
Validation cohort						
KNN	0.60	0.64	0.72	0.56	0.41	0.82
Linear SVM	0.64	0.62	0.38	0.74	0.39	0.74
RBF SVM	0.61	0.68	0.59	0.62	0.40	0.78
GP	0.74	0.79	0.74	0.73	0.55	0.87
RF	0.77	0.88	0.74	0.78	0.59	0.88
MLP	0.73	0.75	0.67	0.76	0.54	0.84
AdaBoost	0.67	0.67	0.67	0.67	0.46	0.82
Naïve Bayes	0.52	0.63	0.77	0.41	0.36	0.80

Note: K-nearest neighbours (KNN); linear support vector machine (linear SVM); radial basis function support vector machine (RBF SVM); Gaussian process (GP); random forest (RF); multilayer perceptron (MLP); adaptive boosting (AdaBoost).
